# Supplementary material for: KSHV transactivator-derived small peptide traps coactivators to attenuate MYC and inhibits leukemia and lymphoma cell growth
Source: Commun Biol. 2021 Dec 2;4:1330. doi: 10.1038/s42003-021-02853-0 (PMC8639922; doi:10.1038/s42003-021-02853-0)
Supplement: Supplementary file 2 — Supplementary Information [file 42003_2021_2853_MOESM2_ESM.pdf]

KSHV transactivator-derived small peptide traps coactivators to attenuate MYC and inhibits leukemia and lymphoma cell growth

Michiko Shimoda[1, 2, *], Yuanzhi Lyu[1], Kang-Hsin Wang[1], Ashish Kumar[1], Hiroki Miura[1], Joshua F. Meckler[2, 3], Ryan R. Davis[4], Chanikarn Chantarasrivong[5], Chie Izumiya[1], Clifford G. Tepper[2, 6], Ken-ichi Nakajima[1], Joseph Tuscano[2, 3], Gustavo Barisone[2, 3], & Yoshihiro Izumiya[1, 2, 6,*]

[1] Department of Dermatology, School of Medicine, University of California Davis (UC Davis), Sacramento
[2] UC Davis Comprehensive Cancer Center, Sacramento, California USA
[3] Department of Internal Medicine, School of Medicine, UC Davis, Sacramento
[4] Department of Pathology and Laboratory Medicine, School of Medicine, UC Davis, Sacramento
[5] Lifematics Inc. Osaka Japan
[6] Department of Biochemistry and Molecular Medicine, School of Medicine, UC Davis, Sacramento

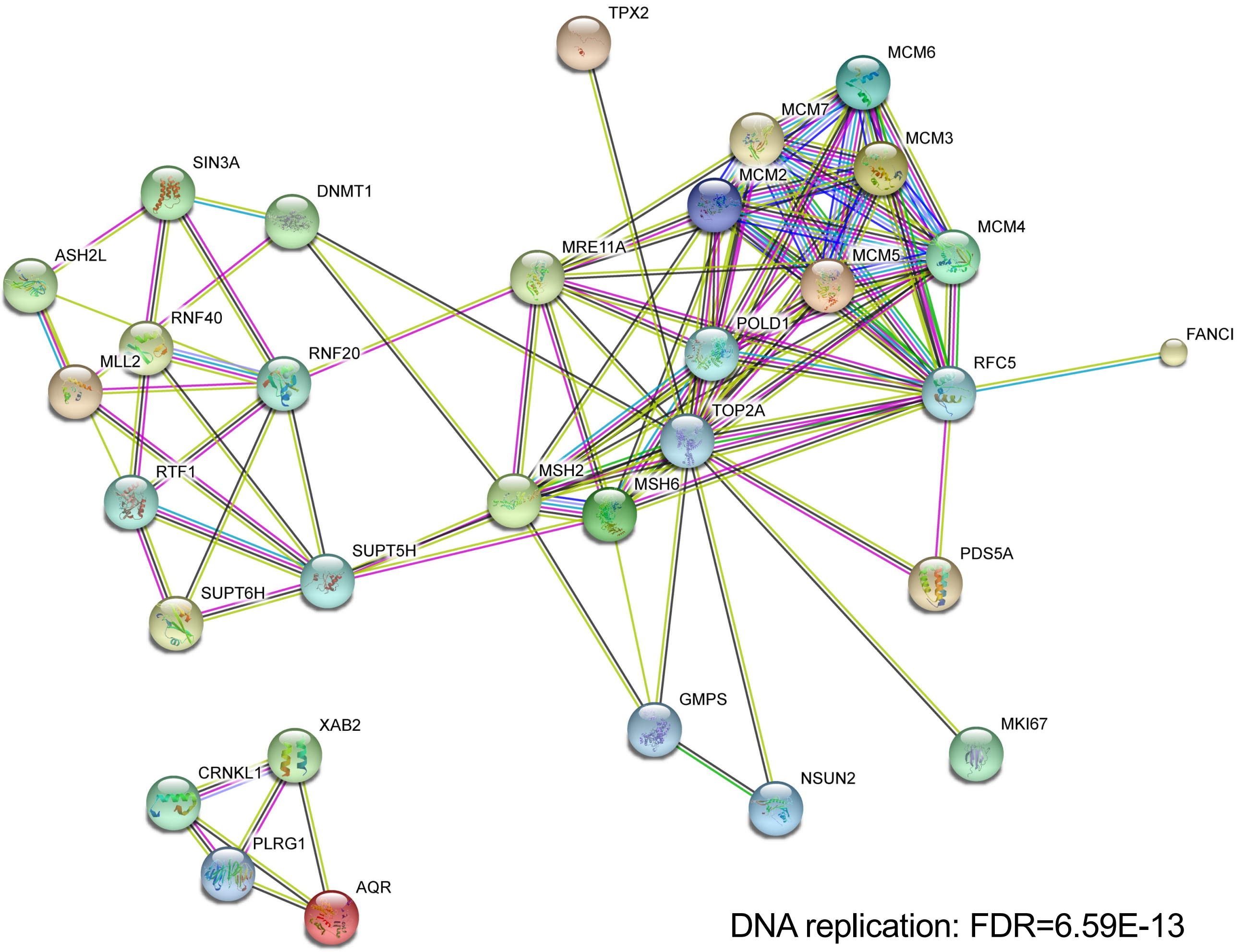

DNA replication: FDR=6.59E-13
DNA mismatch repair: FDR=1.18E-05

S-Figure 1. Protein dissociated from RNAPII during reactivation. Average of peptides counts that are 1.5x less than non-reactivated samples were plotted with STRING.

a

Other herpesvirus & Bacteria	Alignment/protein seq.
KSHV ORF50 (ADQ57929.1):	DDALLSSILQGLYQLDT
LysR Bacteria TF (WP193632878.1):	KAQ-LPDILQGLYQLTQ
Saimiriine GHV2 (CAC85015.1):	DDNILASILQDLYDLPA
Macaca Nemerha Virus (YP010084594.1):	DDDMLAAILQDLYGLQS
Bovine GHV4 (NP076541.1):	EDAYLELILQGLYHLDE
Retrofibro.GHV (YP010084411.1):	DDELLSTILQGLYQLDE
Colobine GHV1 (QDQ69256.1):	DEDLLSAILQGLYQLDE
Rhesus radinovirus (AAF60029.1).	DDDMLAAILQDLYGLQS

b

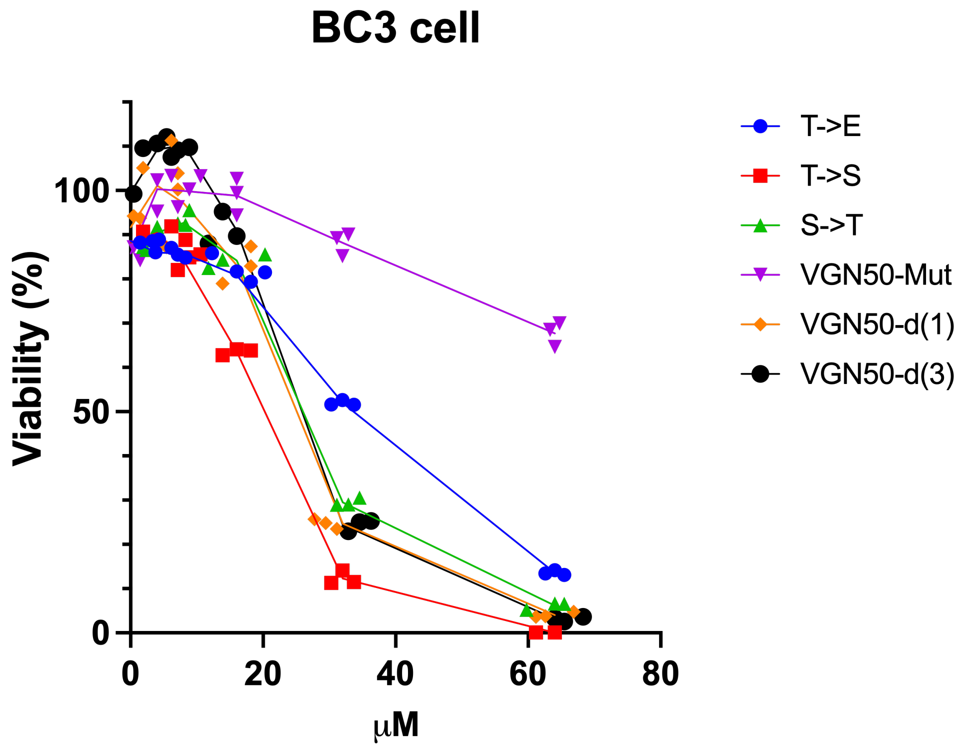

S-Figure 2. (a) Consensus sequences observed at ORF50 transactivation domain with other gamma-herpesviral homologous proteins and a bacterial transcriptional factor. Essential amino acid residue for MYC cell growth inhibition identified in this study is underlined. Conserved amino acid residues are also marked in bold case. **(b) Exchangeable amino acid indicates conserved function with other gamma-herpesvirus family homologous protein.** Amino acids residues that are exchangeable to corresponding amino acid in other herpesvirus without any loss of cell killing in MTT assays were indicated in red (T->E, T->S, S->T). Additionally changing two C-terminal amino acids to D-amino acid (d3) did not increase efficacies of peptide cell killing in vitro.

Supplementary Figure 2

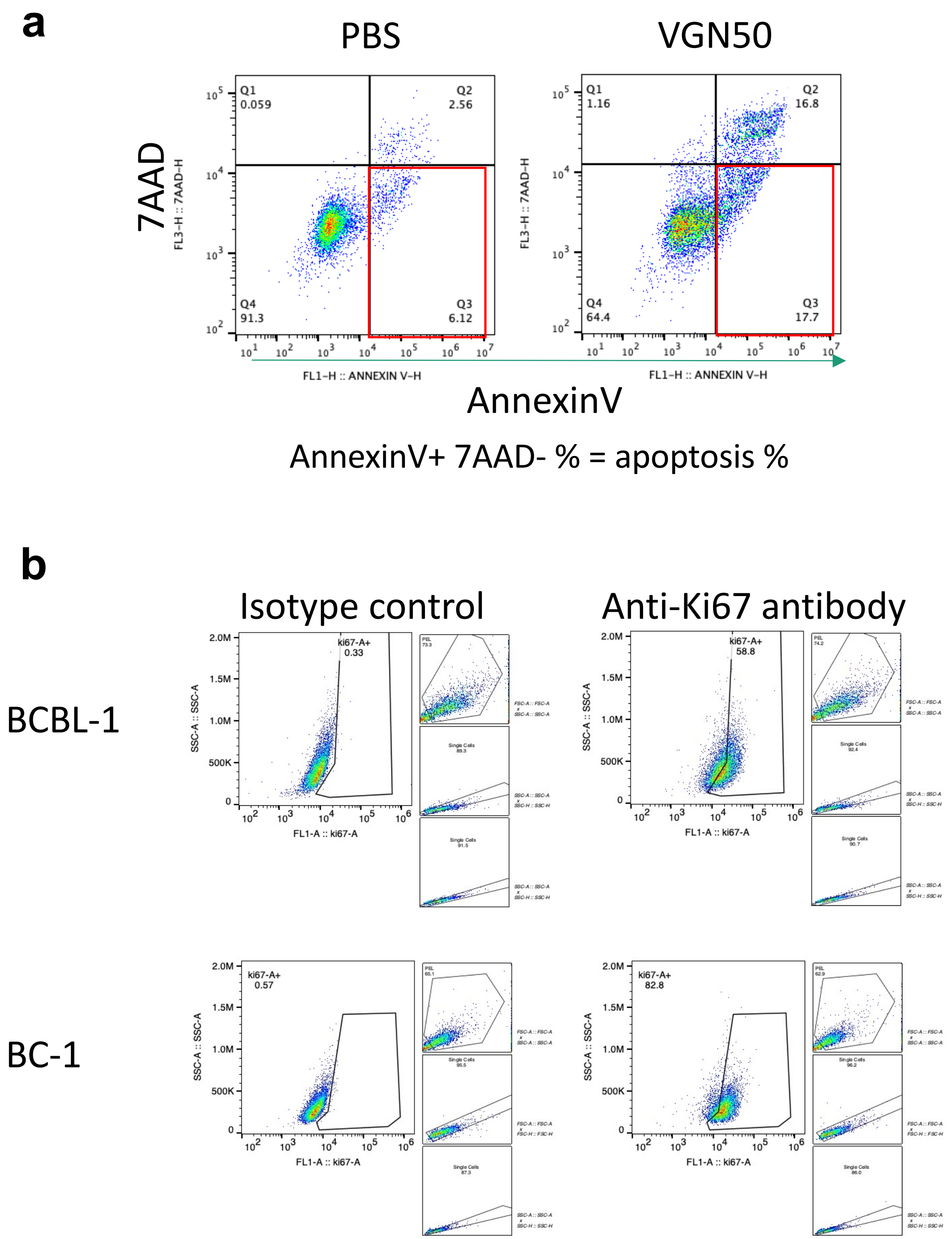

S-Figure 3. Flow cytometry analyses

(a) Apoptosis (%) was determined as AnnexinV+ 7AAD- population gated in red shown in representative FACS plots of BCBL-1 cells treated with PBS or VGN50 at 32μM. (b) Ki67+ (%) gating strategy. Representative FACS plots with ancestry gating strategy of Ki67+ population for BCBL-1 and BC-1 cells treated with PBS with an isotype control are shown. Mean +/-SD of Ki67% of total BCBL-1 and BC-1 cells analyzed are shown in Fig. 2e.

Supplementary Figure 3

a

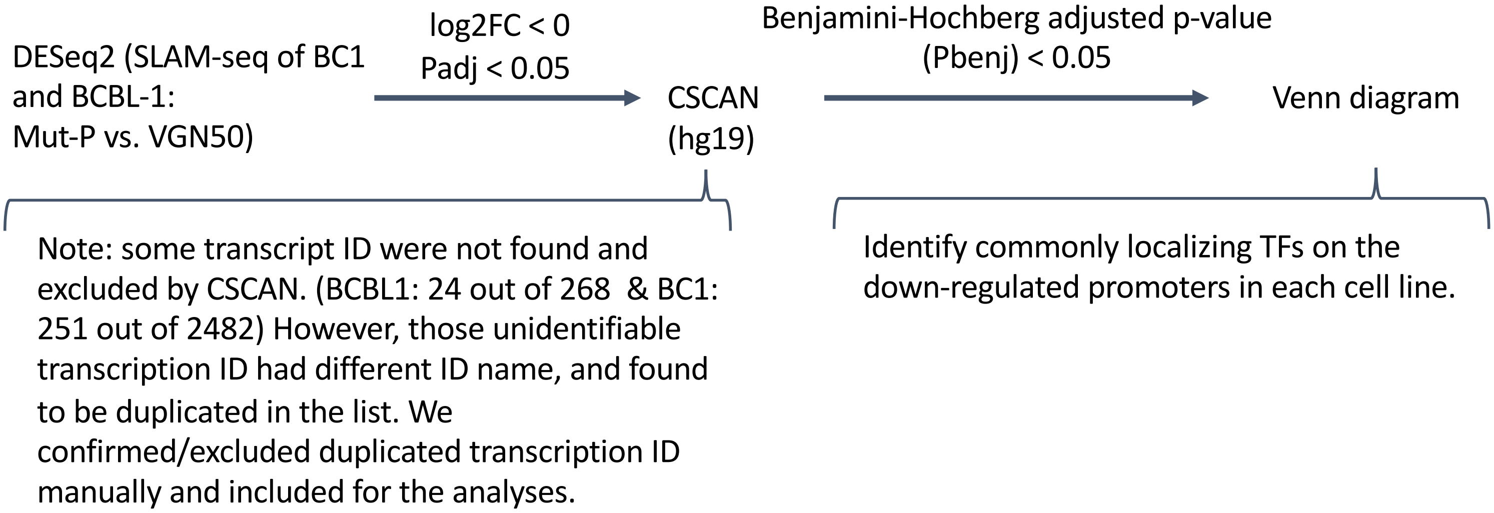

b

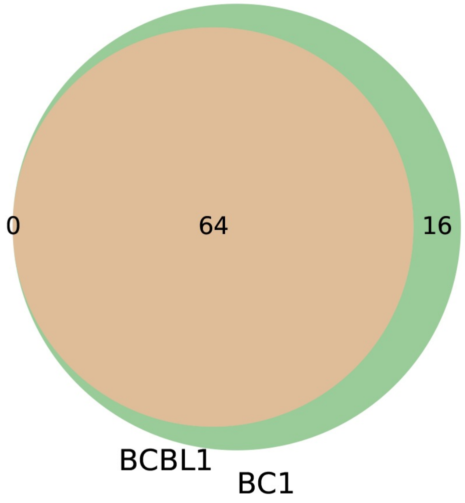

64 transcription regulators in VGN50 down-regulated gene promoters

ETS1	GABP	**p300**
NF-YB	ZEB1	TCF12
ELK1	IRF3	E2F4
BCLAF1	ERRA	Egr-1
CHD2	NF-YA	MEF2A
POU2F2	TCF3	Sin3Ak-20
Mxi1	SRF	ZBTB33
CDP	Nrf1	COREST
c-Fos	ZNF384	SP1
ATF2	BHLHE40	TBP
STAT5A	YY1	USF2
TAF1	MEF2C	Oct-2
**Myc**	RUNX3	PML
BRCA1	TBLR1	MAZ
PAX5-C20	SIN3A	EBF
ELF1	NFIC	RXRA
Pbx3	SREBP1	MTA3
Znf143	EBF1	SIX5
BCL11A	NFATC1	RFX5
**IRF4**	CREB1	PAX5-N19
FOXM1	TR4	NFKB
		CHD1

S-Figure 4. List of putative target transcription regulators of VGN50. (a) Schematic analysis flow for identification of VGN50 targets. Transcription targets were determined by differential transcriptional output between VGN50Mut and VGN50Wt treated cells. After the transcription targets were filtered with indicated cut-off criteria, they were submitted to CSCAN to identify common regulators. The commonly targeted transcription regulators of 2 cell lines were shown as Venn diagram. **(b) Venn diagram and putative VGN50-target transcription regulators.** Number of protein identified is indicated in Venn diagram (left) and list of protein names that are commonly identified between two cell lines were depicted in table (right). Three protein that are known to play important role in PEL cell growth (c-Myc and IRF4) and a protein identified in the targeted coactivator complex (p300 in Fig. 1b) are indicated with bold face.

Supplementary Figure 4

a

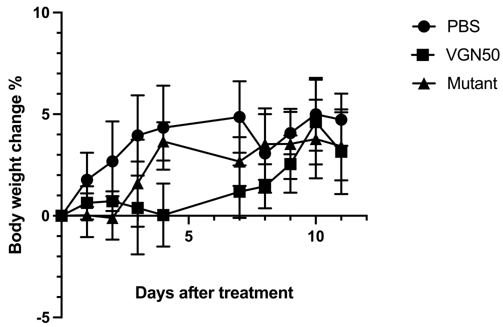

b

	PBS		VGN50 10 MG/KG		Mutant 10 MG/KG		Normal ranges
	AVERAGE	STDEV	AVERAGE	STDEV	AVERAGE	STDEV	
Alanine Transaminase U/L	118.43	101.94	66.45	5.39	82.85	38.16	0-403
Aspartate Transaminase U/L	554.43	183.82	500.33	54.75	651.13	336.08	0-552
Albumin g/dL	3.89	0.11	3.74	0.07	3.67	0.20	2.9-4.0
Alkaline PhosphataseU/L	96.53	7.12	111.00	18.89	129.20	29.09	49-172
Amylase U/L	1789.88	217.38	1669.23	179.42	1624.05	13.13	2463-6660
Blood Urea Nitrogen mg/dL	19.78	1.38	16.43	1.25	20.03	2.53	15.2-34.7
Calcium mg/dL	10.24	0.20	9.00	1.63	9.39	0.60	9.6-11-5
Chloride mmol/L	112.23	1.27	112.88	0.65	112.13	2.05	105-118
Creatinine mg/dL	0.20	0.04	0.25	0.02	0.20	0.06	0.0-0.3
Glucose mg/dL	176.45	6.58	183.45	33.13	194.73	35.16	130-254
Potassium mmol/L	6.59	1.22	5.84	0.75	6.90	0.68	6.9-10.0
Sodium mmol/L	147.50	1.29	147.75	2.50	145.25	1.26	150-160
Phosphorus mg/dL	7.22	1.09	5.83	1.10	7.46	0.95	7.5-10.7
Total Bilirubin mg/dL	0.05	0.02	0.09	0.01	0.05	0.01	0.0-0.2
Total Protein g/dL	5.03	0.21	4.74	0.09	4.81	0.18	4.7-6.1
	AVERAGE	STDEV	AVERAGE	STDEV	AVERAGE	STDEV	
WBC (K/ul)	6.08	2.10	5.87	2.85	4.22	2.04	5.1-14.7
Absolute Neutrophilcells (K/ul)	1.53	0.37	1.81	0.79	1.12	0.66	1.0-3.7
Absolute Lymphocyte cells (K/ul)	3.72	1.47	3.35	1.64	2.54	1.16	4.3-10.6
Absolute Monocyte cells (K/ul)	0.56	0.37	0.49	0.29	0.43	0.12	0.2-1.0
Absolute Eosinophil cells (K/ul)	0.19	0.05	0.16	0.16	0.14	0.06	0.0-0.1
Absolute Basophil cells (K/ul)	0.08	0.05	0.05	0.04	0.06	0.02	0.0-0.1
Neutrophil %	26.04	4.53	32.01	4.24	16.70	14.43	12.5-31.2
Lymphocyte %	60.34	3.27	56.72	4.52	31.21	31.58	62.9-82.7
Monocyte %	8.95	3.20	8.34	1.60	5.52	3.67	2.5-7.5
Eosinophil %	3.36	1.39	2.23	1.57	2.14	0.89	0.0-0.8
Basophil %	1.31	0.34	0.71	0.43	0.70	0.44	0.0-0.5
RBC (M/ul)	8.48	1.41	8.77	0.74	4.85	4.37	8.12-10.55
Hemoglobin (g/dL)	11.85	2.19	13.03	1.23	7.07	6.22	11.7-16.2
Hematocrit %	48.60	8.70	49.38	4.19	27.72	24.63	38.3-54.0
MCV (fL)	57.25	2.04	56.33	0.53	29.04	32.05	45.3-53.6
MCH (pg)	13.98	1.39	14.88	0.25	7.62	7.88	13.3-16.7
MCHC (g/dL)	24.40	1.64	26.38	0.29	13.18	14.14	27.6-32.9
RDW %	19.85	0.66	19.15	0.68	10.08	10.88	12.4-27.0
Platelets (K/uL)	353.75	155.55	524.00	299.84	333.29	152.20	574-1079
MPV (fL)	5.93	0.25	5.43	0.62	3.05	3.04	5.0-20.0

Extended Data Figure 5. BALB/c mice (8 weeks old, female, n=5/group) were injected with 10mg/kg of VGN50 or mutant peptide in 200 uL i.p. with a 5 day-on 2 day off schedule for two weeks. Body weight changes were measured daily (a). At the end of the experiment, fresh blood and serum collected were subjected for complete blood counts and serum biochemical properties, respectively (b).

Supplementary Figure 5

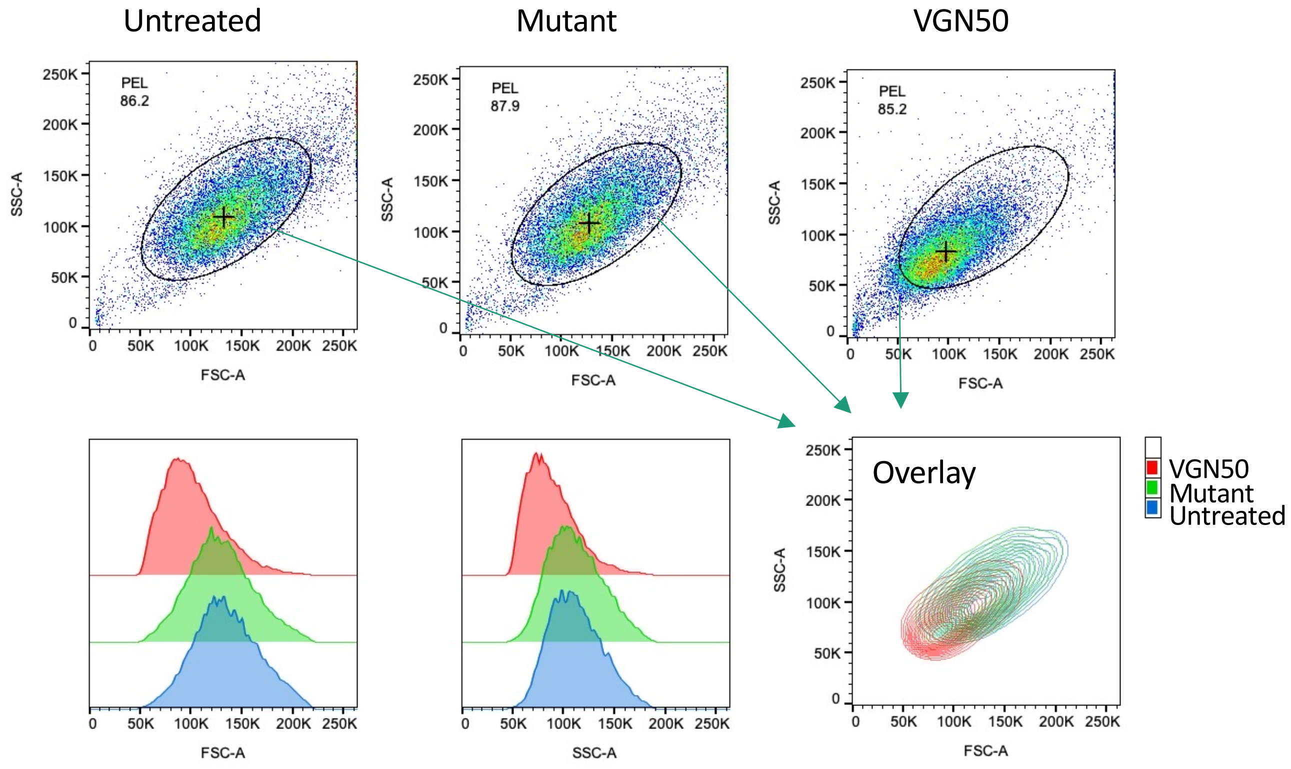

S-Figure 6. Cell atrophy by VGN50. A PEL gate was set based on FSC-A and SSC-A and applied for each ascites sample with a different treatment. A representative overlay FACS plot and histograms for FSC-A and SSC-A are shown. Mean geometric fluorescent intensity (gMFI) +/- SD for FSC-A and SSC-A are shown in Fig. 6C.

Supplementary Figure 6

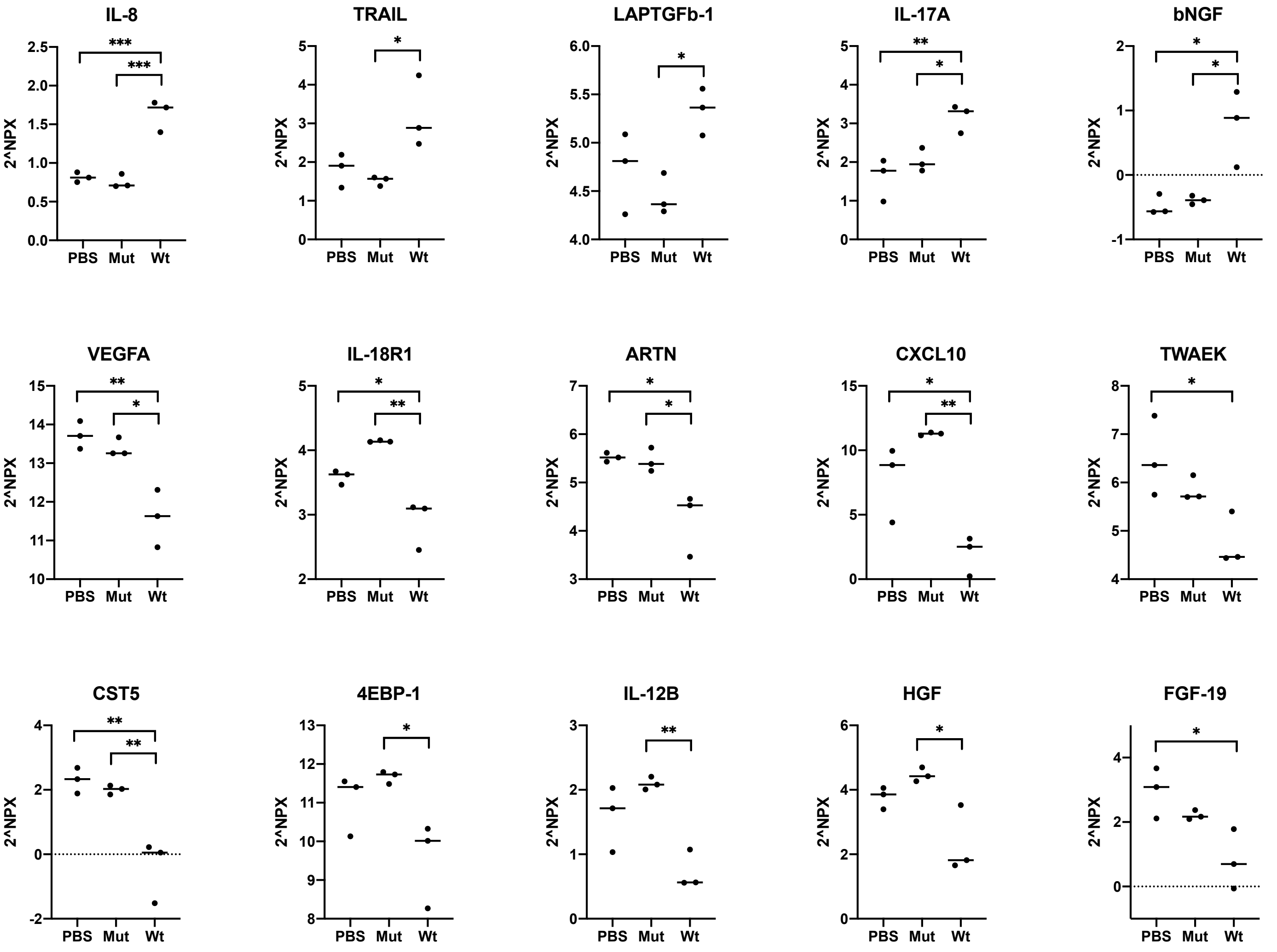

S-Figure 7. Ascites cytokines were measured by Olink assay using inflammatory cytokine panel as shown in STable. Among 92 cytokines, 15 cytokines significantly changed between the groups are shown. * <0.05, ** <0.01, *** <0.001.

Supplementary Figure 7

	GS	NES	NOM p-val	FDR q-val
1	HALLMARK_TNFA_SIGNALING_VIA_NFKB	1.74	0.001	0.034
2	HALLMARK_ESTROGEN_RESPONSE_EARLY	1.69	0.001	0.036
3	HALLMARK_G2M_CHECKPOINT	1.58	0.000	0.068
4	HALLMARK_MITOTIC_SPINDLE	1.57	0.002	0.060
5	HALLMARK_ALLOGRAFT_REJECTION	1.55	0.016	0.061
6	HALLMARK_APOPTOSIS	1.54	0.015	0.055
7	HALLMARK_E2F_TARGETS	1.51	0.001	0.066
8	HALLMARK_INTERFERON_GAMMA_RESPONSE	1.48	0.021	0.071
9	HALLMARK_ESTROGEN_RESPONSE_LATE	1.47	0.020	0.068
10	HALLMARK_IL2_STAT5_SIGNALING	1.39	0.065	0.128
11	HALLMARK_MTORC1_SIGNALING	1.37	0.048	0.137
12	HALLMARK_MYC_TARGETS_V1	1.36	0.053	0.138

S-Figure 8. Total RNA-seq of long-term treated xenograft BCBL-1 cells. Xenograft BCBL-1 cells were isolated from ascites fluid and total RNA was extracted and sequenced. Results of GSEA over mutant peptide treated samples is presented in Table (N=3/group).

Fig. 1c

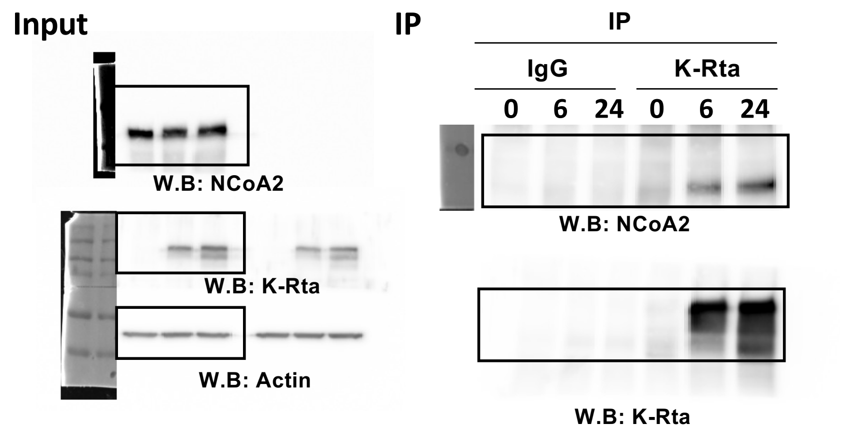

Fig. 1g

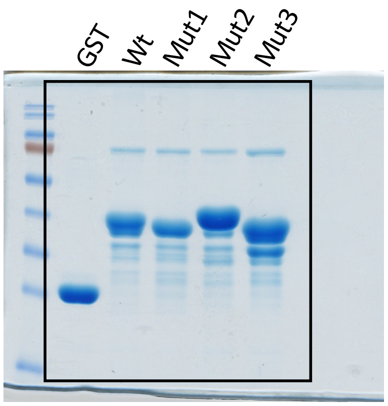

Fig. 1g

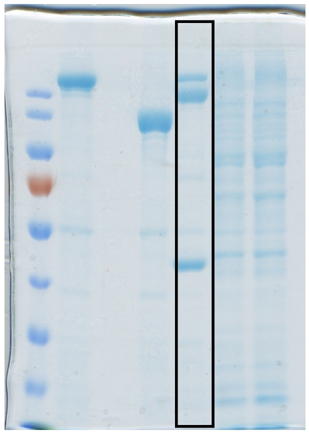

Fig. 1h

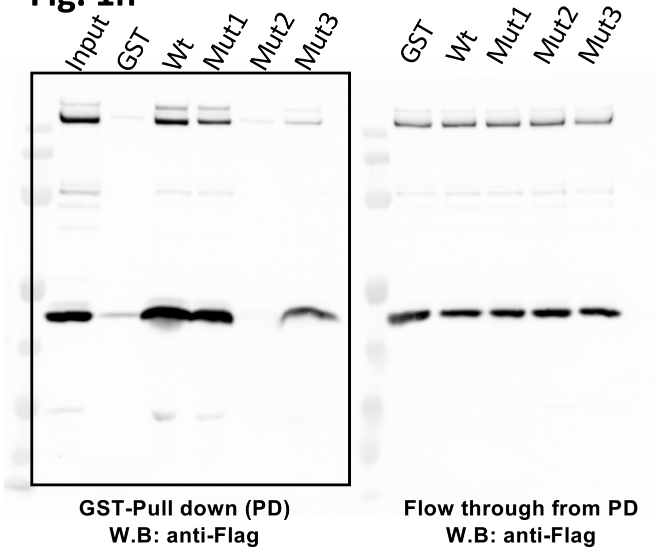

Fig. 3b

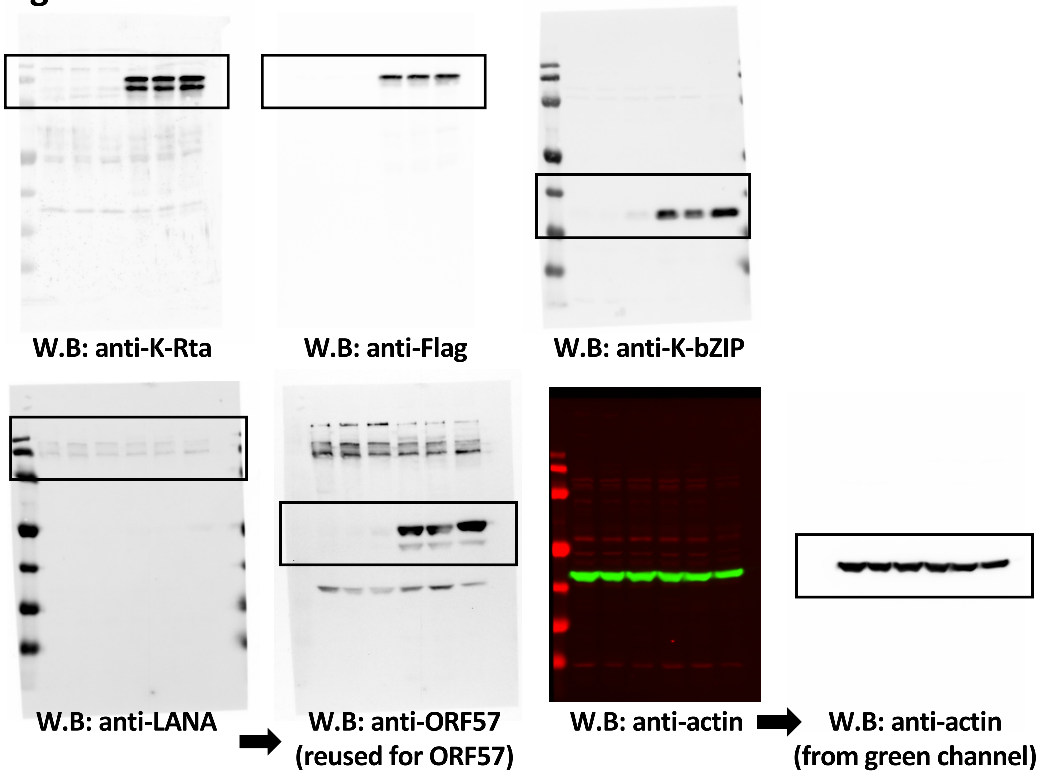

Fig. 5d

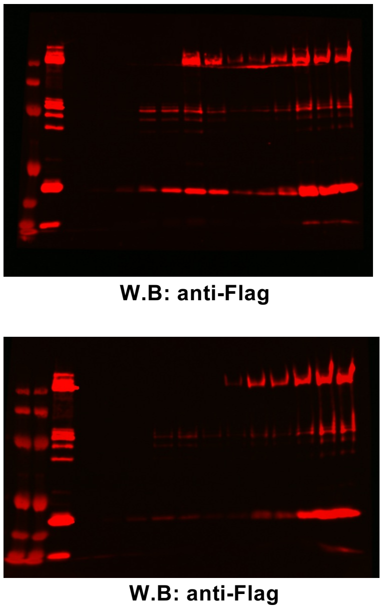

Supplementary Figure 9
