# Supplementary material for: KSHV transactivator-derived small peptide traps coactivators to attenuate MYC and inhibits leukemia and lymphoma cell growth
Source: Commun Biol. 2021 Dec 2;4:1330. doi: 10.1038/s42003-021-02853-0 (PMC8639922; doi:10.1038/s42003-021-02853-0)
Supplement: Supplementary file 3 — Description of Additional Supplementary Files [file 42003_2021_2853_MOESM3_ESM.pdf]

Description of Additional Supplementary Files

File name: Supplementary Data 1

Description: RIME Data sets.

File name: Supplementary Data 2

Description: BCBL-1 SLAM-seq DEseq down regulated p-adj<0.05 and -2 fold.

File name: Supplementary Data 3

Description: BC-1 SLAM-seq DEseq down regulated p-adj<0.01 and -2 fold.

File name: Supplementary Data 4

Description: BCBL-1&BC-1 SLAM-seq DEseq Upregulated, p-adj<0.05, 2-fold.

File name: Supplementary Data 5

Description: Cytokine list.

File name: Supplementary Data 6

Description: Primer sequences.

File name: Supplementary Data 7

Description: Source data for graph and chart presented in main figures.
