# Supplementary material for: KSHV transactivator-derived small peptide traps coactivators to attenuate MYC and inhibits leukemia and lymphoma cell growth
Source: Commun Biol. 2021 Dec 2;4:1330. doi: 10.1038/s42003-021-02853-0 (PMC8639922; doi:10.1038/s42003-021-02853-0)
Supplement: Supplementary file 11 — Reporting Summary [file 42003_2021_2853_MOESM11_ESM.pdf]

nature research

Corresponding author(s): Michiko Shimoda, Yoshihiro Izumiya

Last updated by author(s): 6/07/2021

Reporting Summary

Nature Research wishes to improve the reproducibility of the work that we publish. This form provides structure for consistency and transparency in reporting. For further information on Nature Research policies, see our Editorial Policies and the Editorial Policy Checklist.

Statistics

For all statistical analyses, confirm that the following items are present in the figure legend, table legend, main text, or Methods section.

n/a	Confirmed	
☐	☒	The exact sample size (*n*) for each experimental group/condition, given as a discrete number and unit of measurement
☐	☒	A statement on whether measurements were taken from distinct samples or whether the same sample was measured repeatedly
☒	☐	The statistical test(s) used AND whether they are one- or two-sided<br>*Only common tests should be described solely by name; describe more complex techniques in the Methods section.*
☒	☐	A description of all covariates tested
☒	☐	A description of any assumptions or corrections, such as tests of normality and adjustment for multiple comparisons
☐	☒	A full description of the statistical parameters including central tendency (e.g. means) or other basic estimates (e.g. regression coefficient) AND variation (e.g. standard deviation) or associated estimates of uncertainty (e.g. confidence intervals)
☒	☐	For null hypothesis testing, the test statistic (e.g. *F*, *t*, *r*) with confidence intervals, effect sizes, degrees of freedom and *P* value noted<br>*Give P values as exact values whenever suitable.*
☒	☐	For Bayesian analysis, information on the choice of priors and Markov chain Monte Carlo settings
☒	☐	For hierarchical and complex designs, identification of the appropriate level for tests and full reporting of outcomes
☒	☐	Estimates of effect sizes (e.g. Cohen's *d*, Pearson's *r*), indicating how they were calculated

Our web collection on statistics for biologists contains articles on many of the points above.

Software and code

Policy information about availability of computer code

Data collection

Base calling and quality scoring of sequencing data were performed using Real-Time Analysis software (RTA2.7.7) on the Illumina HiSeq 4000 and with RTA3 on the Illlumina NovaSeq 6000.

Data analysis

CUT&RUN: CUT&RUN sequence reads were aligned to the GRCh38 (hg38) human reference genome and KSHV reference genome (Human herpesvirus 8 strain: GQ994935.1) with Bowtie2. MACS2 was used for detecting peaks following the developer's manual. The default settings with a minimum FDR (q-value) cut-off of 0.05 were used.

RNA-Seq: RNA-Seq data was analyzed using a Salmon-tximport pipeline. Raw sequence reads (FASTQ format) were mapped to the reference human genome assembly (GRCh38/hg38, GENCODE release 36) and quantified with Salmon (DOI: 10.1038/nmeth.4197). Gene-level counts were imported with tximport (Soneson, Love, and Robinson 2015; DOI: 10.12688/f1000research.7563.2). Processed data is a data matrix generated from the output of Salmon and and imported wih tximport. GENCODE release 36 was used for annotation.

SLAM-seq: SLAM-Seq datasets were analyzed using the T>C conversion-aware SLAM-DUNK (Digital Unmasking of Nucleotide conversion-containing k-mers) pipeline utilizing the default parameters (DOI: 10.1038/nmeth.4435, DOI: 10.1186/s12859-019-2849-7). Briefly, nucleotide conversion-aware read mapping of adapter- and poly(A)-trimmed sequences to the human GRCh38/hg38 reference genome assembly was performed with NextGenMap (DOI:10.1093/bioinformatics/btt468). Alignments were filtered for those with a minimum identity of 95% and minimum of 50% of the read bases mapped. For multi-mappers, ambiguous reads and non-3' UTR alignments were discarded, while one read was randomly selected from multimappers aligned to the same 3' UTR. SNP calling (coverage cut-off of 10X and variant fraction cut-off of 0.8) with VarScan2 (DOI: 10.1101/gr.129684.111) was performed in order to mask actual T>C SNPs prior. Non-SNP T>C conversion events were then counted and the fraction of labeled transcripts determined. All output is contained in tcount files.

For manuscripts utilizing custom algorithms or software that are central to the research but not yet described in published literature, software must be made available to editors and reviewers. We strongly encourage code deposition in a community repository (e.g. GitHub). See the Nature Research guidelines for submitting code & software for further information.

Data

Policy information about availability of data

All manuscripts must include a data availability statement. This statement should provide the following information, where applicable:

- Accession codes, unique identifiers, or web links for publicly available datasets
- A list of figures that have associated raw data
- A description of any restrictions on data availability

Data can be accessed via the Source Data files and Supplementary tables. The genomic data discussed in this publication (Figures 3a, 3d, 4a, 5a) have been deposited in NCBI's GEO Database under the SuperSeries GSE173725, which includes 4 SubSeries with accession numbers GSE173724 (CUT&RUN), GSE174031 (RNA-seq for cell culture studies), GSE174033 (RNAseq for PDX study), and GSE174115 (SLAM-seq). Custom codes used to analyze data are available upon reasonable request. Processed data sets were provided as supplemental Tables.

Field-specific reporting

Please select the one below that is the best fit for your research. If you are not sure, read the appropriate sections before making your selection.

☒ Life sciences ☐ Behavioural & social sciences ☐ Ecological, evolutionary & environmental sciences

For a reference copy of the document with all sections, see nature.com/documents/nr-reporting-summary-flat.pdf

Life sciences study design

All studies must disclose on these points even when the disclosure is negative.

Sample size	We did not use statistical methods to predetermine sample sizes. Sample size was determined to be adequate based on the consistency of measurable differences between groups and also prior studies. All biochemical experiments (e.g. binding studies) were replicated more than twice; duplicate biological replicates are considered adequate for ChIP-Seq and CUT&RUN studies according to ENCODE guidelines.
Data exclusions	No data was excluded from analyses.
Replication	All experiments were replicated at least twice and validated repeat experiments.
Randomization	N/A
Blinding	Investigators were not blinded during data acquisition or analyses. It is not common practice for the method we used.

Reporting for specific materials, systems and methods

We require information from authors about some types of materials, experimental systems and methods used in many studies. Here, indicate whether each material, system or method listed is relevant to your study. If you are not sure if a list item applies to your research, read the appropriate section before selecting a response.

Materials & experimental systems

n/a	Involved in the study	
☐	☒	Antibodies
☐	☒	Eukaryotic cell lines
☒	☐	Palaeontology and archaeology
☒	☐	Animals and other organisms
☒	☐	Human research participants
☒	☐	Clinical data
☒	☐	Dual use research of concern

Methods

n/a	Involved in the study	
☐	☒	ChIP-seq
☐	☒	Flow cytometry
☒	☐	MRI-based neuroimaging

Antibodies

Antibodies used	CUT&RUN: RNA Pol II (Millipore-Sigma, CTD4H8), H3K27ac (CST, D5E4), H3K4me3 (CST, C42D8), H3K4me1 (CST, D1A9). Immunoprecipitation and Western blot: primary: LANA (Millipore-Sigma, clone LN53), ORF57 (Santa Cruz Biotech, sc-135747), K8a (Santa Cruz Biotech, sc-57889), K-Rta (Izumiya lab), FLAG (Millipore-Sigma, clone M2), Actin (Millipore-Sigma, AC-15); secondary: anti-mouse IgG-HRP (Thermo Fisher, A28177), anti-rabbit-IgG-HRP (Thermo Fisher, 31460), anti-rat IgG-HRP (Santa Cruz Biotech, sc-2006) Immunofluorescence: RNA Pol II (Millipore-Sigma, CTD4H8), SMARCC1 (CST D7F8S), SMARCA4 (EpiCypher, 13-2002), anti-mouse IgG-Alexa 488 (Thermo Fisher, A11029), anti-mouse IgG-Alexa 647 (Thermo Fisher, A28181), anti-rabbit IgG-Alexa 488 (Thermo Fisher, A11034), anti-rabbit IgG-Alexa 555 (Thermo Fisher, A21430), anti-rabbit IgG-Alexa 647 (Thermo Fisher, A21245).

Validation	Western blotting: correct size of detected bands based on the protein marker. Detection of purified proteins with the antibody. Antibodies used for CUT&RUN were first tested with WB and IFA, and similar studies has been performed by other groups with the antibodies.

Eukaryotic cell lines

Policy information about cell lines

Cell line source(s)	BCBL-1 cels were kindly provided by Dr. Ganem (UCSF). BC-1 (CRL-2230), BC-3 (CRL-2277), BC-1, THP-1 (TIB-202), A549 (CCL-185), Ramos (CRL-1596), HH (CVCL-1280), and U937 (CRL-1593), SU-DHL10 (CRL-2963) were purchased from ATCC. 293FT cell line (R70007) was obtained from Thermo Fisher.
Authentication	Authentication of BCBL-1 was not performed in our laboratory. Purchased cells from ATCC were only used in early passaged cells.
Mycoplasma contamination	Cell lines were tested and found negative for mycoplasma infection.
Commonly misidentified lines (See ICLAC register)	No commonly misidentified lines were used. All cells displayed homologous morphology.

ChIP-seq

Data deposition

☒ Confirm that both raw and final processed data have been deposited in a public database such as GEO.

☒ Confirm that you have deposited or provided access to graph files (e.g. BED files) for the called peaks.

Data access links *May remain private before publication.*	The GEO submission for this study has been approved and both the RNA-seq and CUT&RUN datasets have been unified under SuperSeries GSE173724.
Files in database submission	*Provide a list of all files available in the database submission.*
Genome browser session (e.g. UCSC)	*Provide a link to an anonymized genome browser session for "Initial submission" and "Revised version" documents only, to enable peer review. Write "no longer applicable" for "Final submission" documents.*

Methodology

Replicates	Each CUT&RUN were performed on biological duplicate samples and also performed on two other PEL cell lines to confirm the results.
Sequencing depth	All CUT&RUN libraries were sequenced to achieve 20-30 million mapped paired-end reads.
Antibodies	RNA Pol II (Millipore-Sigma, 05-623, clone CTD4H8, lot 3286043)<br>H3K27ac (CST, 8173, clone D5E4, lot 8)<br>H3K4me1 (CST, D1A9)<br>BRD4 (CST, E2A7X)<br>SMARCC1 (CST, D7F8S)<br>SMARCA4 (EpiCypher, 13-2002)
Peak calling parameters	Paired-end sequencing reads were trimmed using fastp to remove adapter contamination and were then aligned to human hg38 reference genome using Bowtie2 with these options: "--local --very-sensitive-local --no-unal --no-mixed --no-discordant --phred33 -I 10 -X 700". Peaks were called using MACS2 at a q value cutoff of 0.05 using the narrow peak setting.
Data quality	CUT&RUN produced very low backgrounds and specific peaks for histone modification, which was consistent with previously reported peaks.<br>IgG was used as negative control to measure background signals, which is provided in main figure (Fig. 5a).
Software	*Describe the software used to collect and analyze the ChIP-seq data. For custom code that has been deposited into a community repository, provide accession details.*

Flow Cytometry

Plots

Confirm that:

- [x] The axis labels state the marker and fluorochrome used (e.g. CD4-FITC).
- [x] The axis scales are clearly visible. Include numbers along axes only for bottom left plot of group (a 'group' is an analysis of identical markers).
- [x] All plots are contour plots with outliers or pseudocolor plots.
- [x] A numerical value for number of cells or percentage (with statistics) is provided.

Methodology

Sample preparation	Samples for flow cytometry analysis in this study include in vitro cultured primary peripheral blood mononuclear lymphocytes (PBMC), cancer cell lines, and BCBL-1 cells recovered from ascites of xenograft mice. PBMC from healthy donors were prepared from leukapheresis chambers purchased from Vitalant by a density gradient method using lymphocyte separation medium (Lonza). Briefly, leukapheresis samples (~12 ml) will be diluted with 3-fold volume of PBS without Ca and Mg and layered on 12.5 ml of lymphocyte separation medium in a 50ml tube and centrifuged at 400 x g for 20 min at room temperature without break. After removing the top plasma layer, the PBMC layer above clear Ficoll layer was recovered, washed twice with 50ml PBS without Ca and Mg, and then suspended in 10%FBS, RPMI1640 medium for peptide treatment. Cell lines (BCBL-1 and THP-1) were maintained in 15%FBS, RPMI1640 medium, measured their viability (>90%) by trypan blue staining with Countess II and used for peptide treatment. PBMC and cancer cell lines were cultured at 2 x 106/ml in 96 well plate (200l/well), and treated with various indicated concentrations of VGN50 peptide, mutant control peptide, or PBS (solvent) alone for various indicated time in triplicates. Cells were collected by centrifugation at 400 x g and then washed with PBS buffer (200l/well) before staining with live/dead red (Invitrogen) (50l/well) at dark for 30 min according to the manufacturer's protocol, washed by adding FACS buffer (1% FBS/PBS, 200l/well), and resuspended in 200l 2% paraformaldehyde in PBS for 20 minutes at room temperature in dark, washed and resuspended in 200l FACS buffer for flow cytometry analysis. BCBL-1 cells (20-100ul) recovered from ascites of xenograft mice were washed with 1ml PBS by centrifugation at 400 x g for 5 minutes, and then re-suspended in 200ul FACS Buffer, filtered through 40M mesh for flow cytometry analysis of the cellularity.
Instrument	BD Accuri C6, BD Fortessa
Software	Events were acquired with BD Diva and BD Accuri C6 software for BD Fortessa and BD Accuri, respectively, and the acquired data was analyzed with a FlowJo 10.7.2 software.
Cell population abundance	N/A
Gating strategy	In viability assay, the boundaries between positive and negative populations were determined by using MeOH-treated samples containing dead cells and non-treated live cells, of which viabilities were pre-determined by trypan blue staining in parallel. The frequency of live cells in total events were determined by gating SSC-A vs live (FL-3-A-negative) cells with a FlowJo software. In analysis of BCBL-1 cells from ascites of xenograft mice, the FSC/SSC intensity, which is significantly higher than endogenous mouse cells, and the GFP expression (FL-1) in BCBL-1 cells were used to gate BCBL-1 cells.

- [x] Tick this box to confirm that a figure exemplifying the gating strategy is provided in the Supplementary Information.
